# Defining an Optimized Workflow for Enriching and Analyzing Residual Tumor Populations Using Intracellular Markers

**DOI:** 10.1016/j.jmoldx.2024.01.003

**Published:** 2024-01-26

**Authors:** Eve M. Coulter, Findlay Bewicke-Copley, Maximilian Mossner, Trevor A. Graham, Jude Fitzgibbon, Jessica Okosun

**Affiliations:** ∗ Centre for Haemato-Oncology, Barts Cancer Institute, Queen Mary University of London, London, United Kingdom; † Centre for Evolution and Cancer, The Institute of Cancer Research, London, United Kingdom; ‡ Centre for Genomics and Computational Biology, Barts Cancer Institute, Queen Mary University of London, London, United Kingdom; § AstraZeneca, Waltham, Massachusetts

## Abstract

Tumor relapse is well recognized to arise from treatment-resistant residual populations. Strategies enriching such populations for in-depth downstream analyses focus on tumor-specific surface markers; however, enrichment using intracellular biomarkers remains challenging. Using B-cell lymphoma as an exemplar, we demonstrate feasibility to enrich B-cell lymphoma 2 (BCL2)^high^ populations, a surrogate marker for t(14;18)+ lymphomas, for use in downstream applications. Different fixation protocols were assessed for impact on antibody expression and RNA integrity; glyoxal fixation demonstrated superior results regarding minimal effects on surface and intracellular expression, and RNA quality, compared with alternative fixatives evaluated. Furthermore, t(14;18)+ B cells were effectively detected using intracellular BCL2 overexpression to facilitate tumor cell enrichment. Tumor cell populations were enriched using the cellenONE F1.4 single-cell sorting platform, which detected and dispensed BCL2^high^-expressing cells directly into library preparation reagents for transcriptome analyses. Sorted glyoxal-fixed cells generated good quality sequencing libraries, with high concordance between live and fixed single-cell transcriptomic profiles, discriminating cell populations predominantly on B-cell biology. Overall, we successfully developed a proof-of-concept workflow employing a robust cell preparation protocol for intracellular markers combined with cell enrichment using the cellenONE platform, providing an alternative to droplet-based technologies when cellular input is low or requires prior enrichment to detect rare populations. This workflow has wider prognostic and therapeutic potential to study residual cells in a pan-cancer setting.

## Graphical abstract

**Figure:**
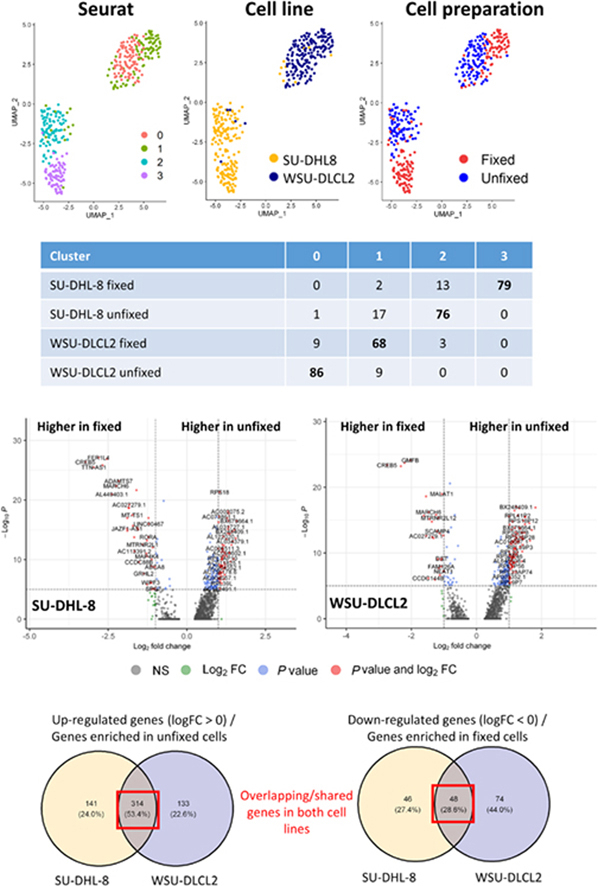

Current therapies targeting biological vulnerabilities in the evolved tumor enable patients to achieve dramatic responses (often resulting in complete remission). However, many patients retain minimal residual disease (MRD).^1^ It is well established that tumor relapses can stem from treatment-resistant residual populations. Therefore, there is a strong impetus to characterize MRD, ideally using single-cell approaches, to unravel our understanding of these rare populations. Numerous techniques exist for monitoring MRD; however, isolation remains a challenge. Historically, these populations are quantified by immunophenotyping and PCR methods or circulating tumor DNA^2^, ^3^, ^4^, ^5^; the former can evaluate individual cells by checking for the presence or absence of specific markers on the cell surface, whereas the latter can identify malignant cells based on characteristic genetic abnormalities (such as mutations and chromosomal changes). Flow cytometric MRD detection can only be considered if there is prior knowledge of tumor-specific surface marker(s) of interest. Furthermore, such populations are often difficult to detect as they are quantified within a heterogeneous sample. Lack of enrichment often makes it difficult to assess whether low-level MRD is due to an absence of disease or due to a depleted cell population following therapy. In contrast, advances in high-throughput single-cell profiling technologies are delivering a better understanding of the complex heterogeneity that exists in both normal and tumor cells. Droplet-based single-cell RNA sequencing can effectively detect transcriptomic differences at the individual cell level, which would inevitably be undetectable by bulk measurement and has led to the identification of tumor-specific markers and cell phenotypes contributing to disease progression, as well as predicting patient response to treatment.^6^, ^7^, ^8^, ^9^, ^10^ Although cost-effective for unbiased high-throughput cell numbers, this strategy, to detect significant numbers of residual tumor cells for analysis, especially following treatment, is often unattainable. Low-throughput platforms, like the plate-based switching mechanism at 5′ end of RNA template sequencing method, have not only been shown to be cost-effective,^11^ but are more compatible for processing lower cell numbers (≤1000 cells per sample) or enriched populations, while providing high-depth sequencing comparable to the droplet-based 10x Chromium (10x Genomics, Pleasanton, CA). The present study showcases a workflow that incorporates a single-cell RNA-sequencing (scRNA-seq) library preparation method (molecular crowding single-cell RNA barcoding and sequencing) with enhanced sensitivity, and reduced reagent costs, when compared with standard single-cell RNA barcoding and sequencing methods (from approximately $1.86 USD (1.70 €) per cell to <$0.59 (<0.54 €) for molecular crowding single-cell RNA barcoding and sequencing).^12^

Despite advances in single-cell sequencing, identification and isolation of residual malignant cells requires tumor-specific surface or intracellular markers that can accurately distinguish normal from malignant cells. Although enrichment based on tumor-specific surface markers is common, the opportunity to achieve this based on the intracellular expression levels of tumor-specific targets has been challenging. To address this, a technique to enrich populations on the basis of an intracellular marker and potential to monitor MRD was developed using a common B-cell lymphoma , follicular lymphoma (FL), characterized by the high intracellular B-cell lymphoma 2 (BCL2) (an anti-apoptosis regulator) expression as an exemplar disease model. In FL, disease progression occurs frequently after treatment with chemo-immunotherapy, and MRD has historically been monitored by quantitative PCR of the *BCL2-IGH* rearrangements, which is currently considered the gold standard in this setting.^13^, ^14^, ^15^, ^16^, ^17^ The technique described within, however, incorporates a cell fixing and permeabilization step, which allows for detection of intracellular biomarkers, in this instance, enriching for tumors with elevated BCL2 expression, a surrogate for *BCL2-IGH* rearrangements, to test the application. The protocol combines real-time, imaged-based, single-cell isolation using the cellenONE F1.4 platform (Cellenion, Lyon, France) with direct scRNA-seq library preparation, thus facilitating the identification, isolation, and phenotypic characterization of these residual populations.

## Materials and Methods

### Cell Line Culture Conditions

Suspension and adherent cell lines were cultured in a 37°C, 5% CO_2_ humidified incubator using RPMI 1640 medium supplemented with 10% fetal bovine serum, 1% l-glutamine, and 1% penicillin-streptomycin. The t(14;18)-positive (WSU-DLCL2) and negative (SU-DHL-8) B-cell lymphoma cell lines, the acute myeloid leukemia cell line (OCI-AML-3), and the breast adenocarcinoma cell line, MCF-7, were all originally acquired from DSMZ (Deutsche Sammlung von Mikroorganismen und Zellkulturen GmbH, The Leibniz Institute DSMZ, Braunschweig, Germany).

### Primary Samples and Ethical Approval (Consent to Participate)

Frozen peripheral blood mononuclear cells (PBMCs) were isolated from one healthy individual (leukocyte cone) and two patients with follicular lymphoma with progressive disease (Supplemental Table S1). Written consent was obtained for the collection and use of specimens for research purposes, with ethical approval obtained from the London Research Ethics Committee of the East London and the City Health authority (10/H0704/65 and 06/Q0605/69). Protocols were conducted in accordance with the Declaration of Helsinki and Good Clinical Practice Guidelines. Clinical information was obtained from the Centre for Haemato-Oncology, Barts Cancer Institute (London, UK) database.

### Comparing Fixative and Permeabilization Protocols and B-Cell Isolation Methods

Four different fixation and permeabilization methods were compared, all commercially available assays and/or published protocols using the fixatives, methanol,^18^ paraformaldehyde in conjunction with the PrimeFLow RNA assay kit (catalog number 88-18005-204; Thermo Fisher Scientific, Waltham, MA) and the split pool ligation-based transcriptome sequencing (SPLiT-seq) fixation method,^19^ and glyoxal (catalog number 50649; Sigma-Aldrich, Burlington, MA; with and without ethanol present in the buffer).^20^ Methods including the use of methanol and paraformaldehyde required further optimization to include the addition of antibody staining steps for enrichment (described in the next paragraph). Cell lines and primary cells were subjected to the different fixation and permeabilization protocols to determine which preparation produced the best quality RNA, for subsequent bulk and scRNA-seq experiments. Fixative solutions were prepared according to the manufacturer's instructions (for the PrimeFLow RNA assay kit), or as previously described (1.33% formaldehyde/5% Triton-X 100^19^ and 3% glyoxal/100% methanol).^20^ The glyoxal solution outlined by Channathodiyil and Houseley^20^ was further modified to omit ethanol, as this improved the quality of the RNA samples.

Furthermore, positive [fluorescence-activated cell sorting (FACS); BD FACSAria Fusion; BD Biosciences; San Jose, CA] and negative (magnetic sorting; Stemcell Technologies, Vancouver, BC, Canada) B-cell isolation strategies were also assessed, to determine which provided the best enrichment method, with minimal impact on the RNA integrity. An additional antibody staining step for surface markers (anti-human CD19/20 AF-647; clones HIB19 and 2H7; catalog numbers 302220 and 302318; anti-human CD3 AF-488; clone HIT3a; catalog number 300320; Biolegend, London, UK) was incorporated before fixing of FL PBMCs, to enable positive B-cell selection by FACS. Similarly, an intracellular staining step to detect BCL2 expression (phycoerythrin; clone Bcl2/100; catalog number A15796; Thermo Fisher Scientific) was also included after permeabilization, in the methods provided by Rosenberg et al^19^ and Channathodiyil and Houseley^20^ (as described in Immunofluorescence Cell Sorting of Primary Samples and Preparation of Cell Line Samples for Single-Cell Isolation). The PrimeFLow assay incorporates fluorescent gene-specific probe sets that target and bind to mRNA.

### Immunofluorescence Cell Sorting of Primary Samples

Healthy and FL patient PBMCs were stained with fluorochrome-conjugated antibodies to CD19/CD20 and CD3 (described in Comparing Fixative and Permeabilization Protocols and B-Cell Isolation Methods) either alone, or in combination with BCL2, following cell fixing and permeabilization. Viable cells were sorted into fractions representing the whole B-cell population (CD19^+^CD20^+^CD3^–^ for experiments determining B-cell isolation method; FL patient samples only), or subpopulations defined by intracellular BCL2 expression [for spike-in experiments confirming an association between elevated BCL2 expression and t(14;18) positivity]. For the latter, FL PBMCs (Patient A only) (Supplemental Table S1) were diluted into healthy PBMCs (at 1% and 0.1% of the PBMC total) and B cells were further sorted into three cohorts, taking cells with the highest, lowest, and intermediate (or medium) BCL2 expression. Each selected subpopulation comprised 10% of the whole B-cell population. A minimum of 10,000 cells were collected per subpopulation into 1.5-mL tubes, and DNA was extracted for major breakpoint region detection by digital-droplet PCR (ddPCR), described in RNA Extraction and Quantification.^21^

### Detection of the Major Breakpoint Region by PCR and ddPCR

The t(14;18) positivity was determined using the ddPCR assay, as previously described.^21^ Briefly, genomic DNA was extracted from a minimum of 100,000 cells using the Qiagen DNA extraction kit (catalog number 69504; Qiagen, Venlo, the Netherlands), and concentration was assessed by Qubit ds assay kit and fluorimeter (catalog number Q32851; Thermo Fisher Scientific). ddPCR was performed with the QX100 droplet digital PCR system (catalog number 186-3001; Bio-Rad Laboratories, Hercules, CA) using 100 ng genomic DNA in a 20 μL master mix (catalog number 186-3010; Bio-Rad Laboratories) containing primers and probes targeting the *BCL2/IGH* translocation; samples were loaded in duplicate. Following droplet generation and PCR, PCR products were loaded into the QX100 droplet reader and analyzed using the QuantaSoft software version 1.7 (Bio-Rad Laboratories). Experiments included a positive control sample [WSU-DLCL2 cell line genomic DNA; t(14:18)+] and a negative control sample [SU-DHL-8 cell line genomic DNA; t(14;18)–].

### RNA Extraction and Quantification

RNA was extracted from bulk live, and fixed and permeabilized, cell lines and FL primary samples (PBMCs and sorted B cells) using the RNeasy plus kit (catalog number 74104; Qiagen, Venlo, the Netherlands), according to the manufacturer's instructions. RNA concentrations were quantified using the Qubit RNA assay kit (high sensitivity; catalog number Q32852; or broad range; catalog number Q10210; Thermo Fisher Scientific), and the quality was assessed using the Agilent 2200 TapeStation system (Agilent Technologies, Santa Clara, CA), to determine RNA integrity number (RIN) scores.

### Quantitative Real-Time PCR of Housekeeping Genes

cDNA was synthesized according to the manufacturer's instructions using the high-capacity cDNA reverse transcription kit (catalog number 4368814; Thermo Fisher Scientific). PCRs were performed using 100 ng total RNA from cell lines and primary B cells (derived from immunomagnetic negative selection), with the following program: 25°C for 10 minutes, 37°C for 2 hours, and 85°C for 5 minutes. The resulting 20 μL of cDNA was diluted to 300 μL in nuclease-free water and stored at –20°C. Housekeeping genes, glyceraldehyde-3-phosphate dehydrogenase (forward: 5′-CCATCACCATCTTCCAGGAG-3′; and reverse: 5′-GAGATGATGACCCTTTTGGC-3′) and 18S (forward: 5′-AAACGGCTACCACATCCAAG-3′; and reverse: 5′-CCTCCAATGGATCCTCGTTA-3′), were assessed to measure the impact of the different fixative conditions on gene expression levels. Real-time quantitative PCR analysis was performed in 384-well plates by mixing 3 μL of cDNA with 7 μL of SYBR green primer master mix [5 μL SsoAdvanced universal SYBR green supermix (catalog number 1725271; Bio-Rad Laboratories), 1.6 μL of nuclease-free water, and 0.2 μL of 10 μmol/L forward and reverse primers] in each well, with samples run in triplicate. Reactions were performed using a PCR melt protocol, as follows: 95°C for 2 minutes, then 39 cycles of 95°C for 5 seconds, 60°C for 30 seconds, followed by 95°C for 5 seconds, 65°C for 5 seconds, and 95°C for 5 seconds on the Bio-Rad CFX384 real-time quantitative PCR machine (Bio-Rad Laboratories). Average raw cycle threshold values (for triplicate samples) were calculated; each symbol from the scatterplot represents an independent experiment (*n* = 3). Data were analyzed with CFX-manager software version 3.1 (Bio-Rad Laboratories).

### Preparation of Primary and Cell Line Samples for Bulk mRNA Library Generation and Sequencing

B cells (from Patient A with FL) were isolated from a maximum of 50 million freeze-thawed PBMCs using the EasySep human B-cell enrichment kit (catalog number 19054; immunomagnetic negative selection kit, yielding untouched and highly purified B cells; Stemcell Technologies). B-cell purity was checked using surface anti-human CD19-allophycocyanin antibody (Biolegend) staining and detection by flow cytometry. RNA was extracted from cell lines, and isolated primary B cells (unfixed and glyoxal fixed, with and without ethanol) were submitted externally (Novogene, Cambridge, UK), for mRNA library preparation (poly-A enrichment) and 150-bp paired-end sequencing on the NovaSeq 6000 instrument.

### Preparation of Cell Line Samples for Single-Cell Isolation

**Figure 1:**
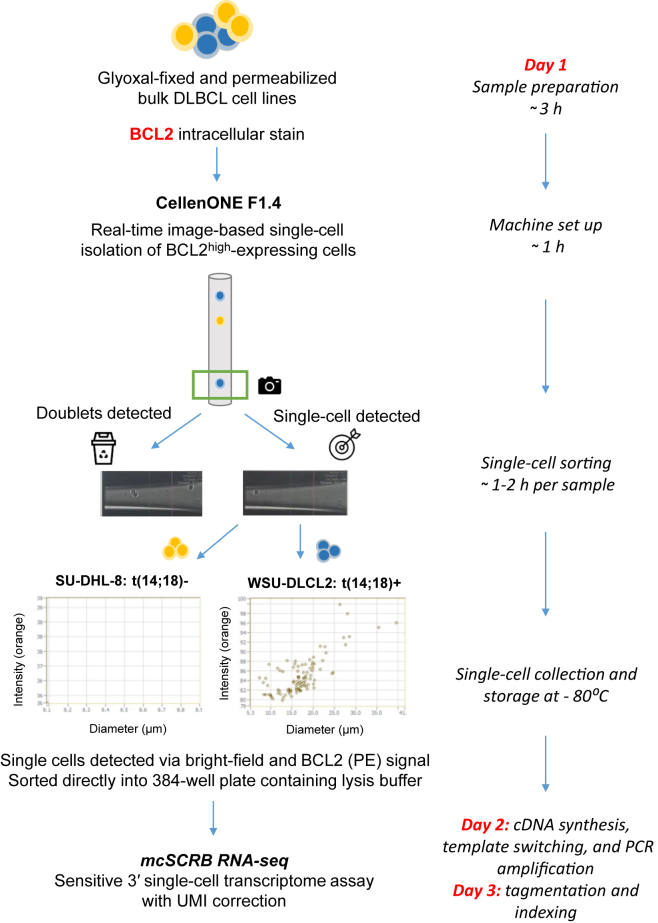
Schematic overview of the experimental workflow for t(14;18)+ single-cell isolation and characterization. Cell lines were fixed with glyoxal (without ethanol) and permeabilized with methanol before intracellular staining for B-cell lymphoma 2 (BCL2) expression. The t(14;18)-positive B cells were isolated following flourescence-based cell sorting using the cellenONE F1.4 single-cell isolation platform, as shown in the cellenONE flourescence intensity plots for WSU-DLCL2 and SU-DHL-8 cell lines (labeled with anti-BCL2 antibody, directly conjugated to phycoerythrin (PE); events were detected and acquired in the orange channel. Single cells were sorted into individual wells of a 384-well plate containing lysis buffer reagents, to initiate the direct single-cell RNA-sequencing library preparation (adapted method from Bagnoli et al^12^). Practically, the workflow can be completed within 72 hours, depending on the number of cells, or plates collected (there is an option to set up automation on the cellenONE F1.4. and run sample collection overnight at 4°C). DLBCL, diffuse large B-cell lymphoma; mcSCRB RNA-seq, molecular crowding single-cell RNA barcoding and sequencing.

For scRNA-seq experiments, serial dilutions were established containing ≤1% WSU-DLCL2 t(14;18)+ cell line in a t(14;18)– SU-DHL-8 background. A maximum of 5 × 10^6^ cells were incubated with a fixable viability dye (eFluor-780; catalog number 65-0865-14; Thermo Fisher Scientific) on ice for 15 minutes, washed with 1× phosphate-buffered saline, then fixed with glyoxal (without ethanol) for a further 15 minutes on ice. Following 30 minutes of permeabilization with 100% methanol on ice, cells were incubated with anti-human BCL2-phycoerythrin antibody (diluted in 1× phosphate-buffered saline/1% bovine serum albumin), and then washed twice with 1× phosphate-buffered saline; the first was supplemented with 1% bovine serum albumin, and the second was supplemented with 0.04% bovine serum albumin. Live (unfixed) and glyoxal-fixed cells were resuspended at a concentration of 0.2 × 10^6^/mL 1× phosphate-buffered saline/0.04% bovine serum albumin, and single cells were sorted immediately into 384-well plates containing lysis buffer, using the cellenONE F1.4 platform (Figure 1). Excess fixed/permeabilized cells were stored for an additional 24 hours at 4°C before single-cell sorting (to assess the effect of increased incubation time on the sequencing read quality).

### Detection and Isolation of t(14;18)+ and t(14;18)– Cells Using the cellenONE F1.4 Platform

Individual cells were isolated using the cellenONE F1.4 platform (Figure 1), an image-based, multiparameter detection and sorting platform that uses piezo-acoustic droplet generation for ultragentle cell isolation. Fluorescence and transmission settings were optimized (according to the manufacturer's F1.4 software user guide) to determine the threshold for BCL2 overexpression (compared with BCL2 endogenous expression) using the t(14;18)+ and t(14;18)– cell lines (Figure 1). Once established, viable t(14;18)+ cells were isolated from serial dilutions overexpressing BCL2, whereas t(14;18)– cells were selected on viability staining alone.

A total of 96 fixed single cells from each cell line were sorted directly into 384-well plates containing lysis buffer, immediately and 24 hours after fixation. The latter time point was selected to assess whether extending the incubation time (between fixing and sorting) would compromise the ability of glyoxal to preserve intact RNA, and ultimately affect the overall quality of the sequencing data. Unfixed single cells (96 from each cell line) were also collected to make a direct comparison with glyoxal without ethanol (G-E) fixed cells, and determine any changes in gene expression as a consequence of cell fixation.

### scRNA-Seq cDNA Synthesis and Library Preparation

Single-cell RNA barcoding and sequencing was performed according to the protocol described by Bagnoli et al.^12^ This modified plate-based protocol includes a higher concentration of polyethylene glycol (catalog number 528877; Sigma-Aldrich, St. Louis, MO) to induce molecular crowding conditions, which, in turn, increased sensitivity and cDNA yield. Briefly, single cells were sorted directly into lysis buffer containing Proteinase K (catalog number 9034; Takara, San Jose, CA) and barcoded oligo-dT primer (E3V6NEXT; IDT, San Diego, CA). Following protein digestion, reverse transcription master mix (containing template-switching oligo; IDT) was added to individual wells. cDNA synthesis and template switching were performed in the presence of 7.5% polyethylene glycol 8000 solution, and the barcoded cDNA was pooled and cleaned using solid-phase reversible immobilization (SPRI) beads (Beckman Coulter Life Sciences, Brea, CA). The final steps of the cDNA synthesis included removal of residual primers, followed by PCR amplification. The quality and quantity of the purified cDNA were assessed (using the Qubit DNA assay kit and Tape Station; Agilent Technologies) before library PCR. Barcoded cDNA generated from both fixed and unfixed single cells was pooled to construct three individual libraries; the first contained cells collected 24 hours after fixing (fixed SU-DHL-8 and WSU-DLCL2 cells only); the second, SU-DHL-8 cells (both fixed and unfixed cells); and the third, WSU-DLCL2 cells (both fixed and unfixed cells). The pooled libraries were then size selected using SPRI beads at a ratio of 1:1 and eluted in 20 μL Tris-EDTA buffer, before Nextera XT (Illumina, San Diego, CA) tagmentation and sequencing (150-bp paired-end sequencing). In total, 192 million reads per library pool were sequenced on an Illumina Novaseq 6000 platform (Novogene).

### Bulk and scRNA-Seq Quality Control and Data Analysis

Quality metrics of the raw FASTQ files were generated using FASTQC version 0.11.9 (*https://www.bioinformatics.babraham.ac.uk/projects/fastqc*, last accessed November 2, 2023). For bulk RNA sequencing (RNA-seq), FASTQ files were aligned to GRCh38 using hisat2 version 2.1.0, and feature counting was performed using htseq-count version 0.13.5.^22^^,^^23^ For scRNA-seq, FASTQ files were processed using zUMIs version 2.9.7.^24^ Bases 1 to 6 of read 1 were the cell barcodes, with bases 7 to 16 used as unique molecular identifiers (UMIs). The first 50 bases of read 2 were the cDNA read that was aligned to the genome. The resulting UMI counts were then analyzed using the Seurat R package version 4.3 (*https://satijalab.org/seurat*). The individual cell barcodes were mapped back to the known barcodes for each well in the 384-well plate, and cells with a barcode not found on the plate were excluded from the analysis. Quality control of the individual cells was performed, and only cells passing the following filters were retained: number of unique transcripts in the cell ≥500, number of genes detected in the cell ≥250, log10 genes per UMI >0.8, and mitochondrial UMIs <20%. Cell cycle scoring was performed using the Seurat CellCycleScoring function, and the difference between the S and G phase scores was calculated. Data were normalized using SCTransform version 2.0.^25^ with the cell cycle score difference and the percentage of mitochondrial genes included in the vars.to.regress argument. A UMAP (Uniform Manifold Approximation and Projection) of the normalized data was constructed, and neighbors were found using 30 principal components. Clusters were found with the resolution set to 0.8. Differentially expressed genes were found using the Seurat FindMarkers function, and pseudobulk counts were generated using the AggregateExpression function.

## Results

### Detection of t(14;18)+ Cells: Specific Cell Fixation and Permeabilization Methods Diminish Surface Expression, but Not Intracellular BCL2 Expression Levels

Fixation and permeabilization conditions were tested, and BCL2 expression levels were evaluated by flow cytometry. Expression levels were compared in two B-cell lymphoma cell lines [WSU-DLCL2, a t(14;18)-positive cell line, and SU-DHL-8, a t(14;18)-negative cell line], to discriminate true BCL2 high signal (or overexpression, resulting from the translocation), in contrast to endogenous expression. BCL2 expression levels were also compared with that of B cells from a FL patient PBMC sample. In all protocols tested, flow cytometry analysis showed clear distinction between cells overexpressing BCL2 in the WSU-DLCL2 cell line compared with background expression in SU-DHL-8 cells at both the mRNA and protein level (Supplemental Figure S1A). FL B cells also demonstrated consistent levels of BCL2 expression across all protocols, similar to the t(14;18)+ cell line, WSU-DLCL2 (Supplemental Figure S1A). Fixation and permeabilization conditions were also assessed on surface protein expression of FL B cells. Variation in the CD19 and CD20 expression levels was observed across all conditions tested (Supplemental Figure S1B). Signal quenching was also detected following methanol fixation, and it was more difficult to discriminate a B-cell population by flow cytometry (Supplemental Figure S1B).

### Glyoxal Improves RNA Integrity and Has No Significant Effect on Bulk mRNA Expression

**Figure 2:**
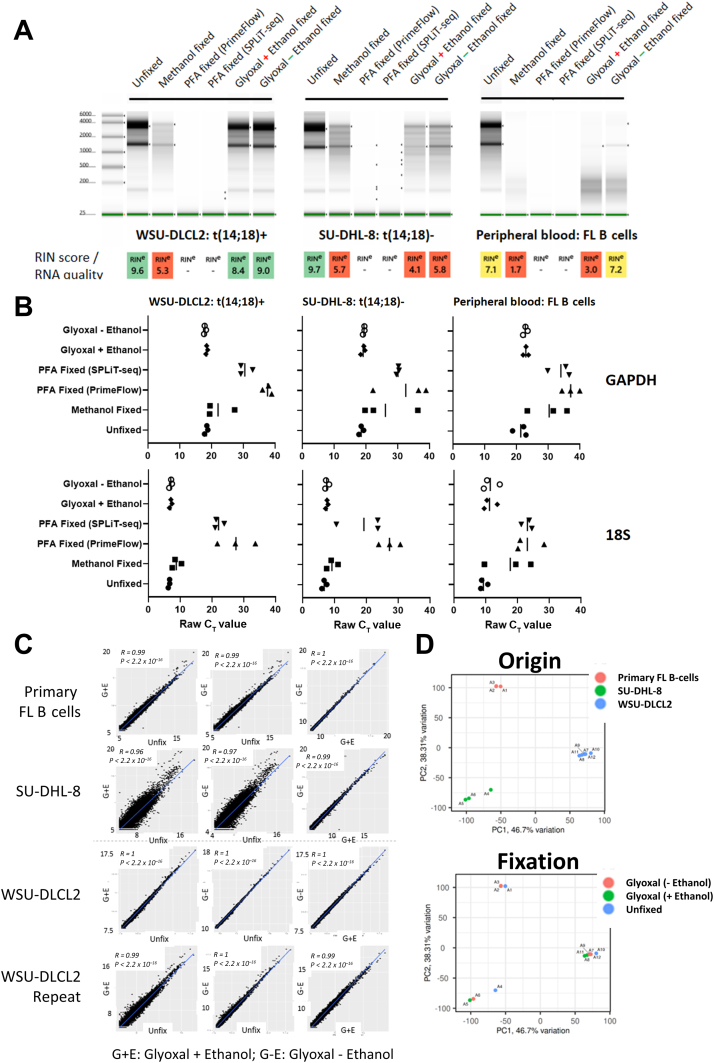
Optimization of cell fixing and permeabilization conditions. **A:** RNA integrity was assessed and scored [RNA integrity number (RIN)] in two B-cell lymphoma cell lines (WSU-DLCL2 and SU-DHL-8), and follicular lymphoma (FL) B cells (isolated from peripheral blood mononuclear cells from Patient A, following negative magnetic sorting), and compared across the different fixation and permeabilization conditions. For glyoxal-fixed samples, + indicates with ethanol and – indicates without ethanol. **B:** Expression levels of two housekeeping genes, glyceraldehyde-3-phosphate dehydrogenase (GAPDH) and 18S (as measured by quantitative real-time PCR), were comparable between cells fixed with glyoxal (with or without ethanol) to unfixed cells. Cycle threshold (C_T_) values for paraformaldehyde (PFA)–treated cells were notably higher than when compared with unfixed cells. **C:** Correlation plots measuring differential gene expression between glyoxal fixed (with or without ethanol) and unfixed primary B cells (**top row**), SU-DHL-8 cells (**second row**), and WSU-DLCL2 cells [**third row**; including a biological repeat (**bottom row**)]. **D:** Principal component (PC) analysis plots displaying sample clustering based on cell origin/biology (**top panel**) and cell preparation method (**bottom panel**). SPLiT-seq, split pool ligation-based transcriptome sequencing.

Despite the marginal impact of these fixatives on surface and intracellular expression (Supplemental Figure S1, A and B), the effect of fixation was more pronounced at the RNA level (Figure 2A). RIN measurements were used to correlate with RNA quality and suitability for downstream gene expression analyses (high RIN = more intact RNA quality). All fixatives tested had an impact on RNA quality; levels of degradation were sample type specific (RIN values were superior in cell lines) (Figure 2A); and for the primary sample, they were dependent on the B-cell enrichment method (RIN scores were lower following FACS compared with negative magnetic sorting of B cells) (Supplemental Figure S1C). Paraformaldehyde had the most profound effect on RNA integrity, and often failed to record a RIN score on both the broad-range and high-sensitivity screen tapes (Figure 2A and Supplemental Figure S1, C–E). RIN scores for fixed samples were greatest when using fixing buffers containing glyoxal, and were improved further when ethanol was removed from the solution (Figure 2A and Supplemental Figure S1, D and E). Despite the lower RIN scores when fixing with glyoxal, the expression levels by quantitative real-time PCR of housekeeping genes (glyceraldehyde-3-phosphate dehydrogenase and 18S) were largely unchanged when compared with unfixed cells (Figure 2B). Moreover, bulk mRNA sequencing analysis revealed that glyoxal (with or without ethanol) had no significant effect on the gene expression profile when compared with unfixed cells in both cell lines and primary FL B cells (Figure 2C). Furthermore, gene expression data from two WSU-DLCL2 replicate experiments comparing unfixed and glyoxal-fixed (without ethanol) samples identified only 48 differentially expressed genes of a total 22,922 genes annotated in Ensembl GRCh38 (Supplemental Table S2). This indicated that few genes were systematically affected by the cell preparation method, and the method did not significantly enrich for any functional category by Gene Ontology analysis. Principal component analysis revealed that samples clustered based on cell type (origin), but not fixation status (Figure 2D). Going forward G-E was taken as the preferred fixative for subsequent single-cell experiments.

### High BCL2 Expression Correlates with t(14;18) Positivity in Glyoxal-Fixed B Cells

Having identified robust fixation and permeabilization conditions that did not compromise RNA quality, the next step was to verify BCL2 overexpression as a unique biomarker to distinguish tumor B cells within a pool of normal B cells. To demonstrate proof of concept, the two B-cell lines were used in a cell mixing experiment, and FACS-sorted bulk BCL2^high^-expressing cells from serial dilutions containing ≤1% WSU-DLCL2 cells in a t(14;18)-negative background (SU-DHL-8). The t(14;18)-positive cells were identified within the BCL2^high^-expressing cell fraction, through detection of the translocation by PCR of the *BCL2* breakpoints in the major breakpoint region of the gene (Supplemental Figure S2A). Experiments were replicated using a primary PBMC sample; FL PBMCs were spiked in to a healthy PBMC sample at 1% and 0.1% of the total PBMC number. CD19^+^CD20^+^CD3^–^ B cells were FACS sorted into subpopulations based on high, intermediate (medium), and low BCL2 expression, and a minimum of 10,000 cells from each sub-fraction were collected for DNA extraction and confirmation of t(14;18) positivity. ddPCR demonstrated an enrichment for t(14;18)+ cells in BCL2^high^-expressing B cells compared with cellular fractions expressing intermediate and low levels of BCL2 (Supplemental Figure S2B).

### Direct Harvesting of Glyoxal-Fixed Single Cells Generates Good Quality Sequencing Libraries

**Figure 3:**
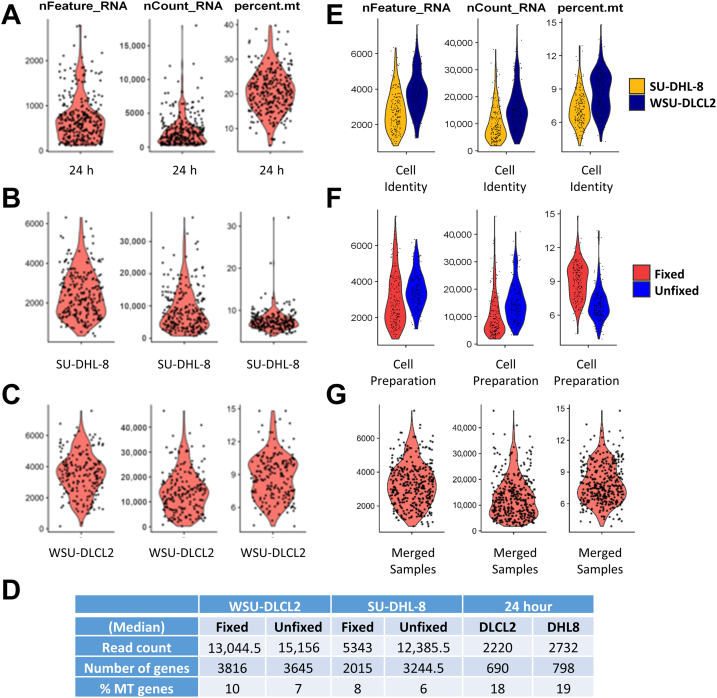
Feature count metrics from single-cell RNA-sequencing data. **A**–**C:** Violin plots featuring the number of genes/cell, reads/cell, and percentage mitochondrial (MT) genes for both cell lines analyzed 24 hours after fixing and permeabilizing cells (stored at 4°C; **A**) and libraries generated from fixed and unfixed cells immediately after sorting for SUDHL-8 cell line (**B**) and WSU-DLCL2 cell line (**C**). **D:** Median read counts, number of genes, and percentage mitochondrial genes present for individual cell lines, sample preparation, and time points, outlined in **A**–**C**. Because of the poor quality control (QC) results from the 24-hour post-fixation libraries, these data were excluded from further analysis. **E**–**G:** No significant differences were detected when comparing QC metrics between individual cell lines (**E**) and fixed versus unfixed cells when cell lines were combined (**F**) and were combined for subsequent analysis (**G**).

To investigate the effect of glyoxal (G-E) on gene expression at the single-cell level, a modified RNA-seq library generation protocol^12^ was used to obtain transcriptomes from single cells isolated using the cellenONE F1.4 platform (Figure 1). Single-cell RNA-sequencing analysis was performed following quality filtering and batch correction using Seurat. Initial quality control analysis identified that libraries generated from cells sorted 24 hours after fixation (stored at 4⁰C) expressed a lower percentage of genes and reads per cell. In addition, the percentage of mitochondrial genes was also higher compared with libraries generated from cell lines immediately after sorting (Figure 3, A–D). The authors hypothesized that the extended incubation time before sorting into lysis buffer reagent may have led to cell membrane rupturing, thus resulting in the higher proportion of mitochondrial genes. The 24-hour post-fixation libraries were therefore excluded from further analysis. For the remaining libraries, no substantial differences were observed in the quality control metrics between the two cell lines (Figure 3E) and, specifically, the basal gene expression levels between fixed and unfixed cells (Figure 3F). Subsequently, the two libraries were merged to form a single scRNA-seq data set (Figure 3G). As part of the final quality-filtering process, cells with no assigned (or unrecognizable) barcodes were removed from the analysis. In total, 174 fixed cells and 189 unfixed cells progressed to the analysis pipeline for further investigation.

### Concordance in Single-Cell and Bulk RNA-Seq Analysis Shows Overlapping Gene Expression Profiles

The next step was to compare the gene expression profiles generated from single-cell and bulk RNA-seq analysis. This was to determine whether both platforms could enrich for the same gene signatures, as well as identify those that discriminate between the B-cell biology. Pseudo bulk samples from single-cell RNA counts were generated, and then combined with the raw counts from the bulk mRNA experiment, before data normalization. Correlation plots comparing pseudo bulk and corresponding bulk samples showed equivalent gene expression profiles within each cell line for both live and fixed samples (Supplemental Figure S3A). Furthermore, heat maps generated from the top 100 expressed genes show bulk RNA-seq samples from SU-DHL-8 (A4-6) and WSU-DLCL2 (A7-12) cell lines clustered accordingly with their single-cell counterparts, in both fixed and unfixed conditions (Supplemental Figure S3, B and C, and Supplemental Table S3). These data also highlighted a high degree of similarity between cell preparation conditions, but distinct differences between the cell of origin. Overall, this demonstrates a good correlation between the two independent bulk and single-cell experimental data sets that ultimately enrich for the same gene signatures.

### scRNA-Seq Analysis Discriminates Clusters Predominantly on the B-Cell Biology of the Cell Lines and Reveals Minimal Transcriptomic Changes Between Glyoxal-Fixed (G-E) and Unfixed Cells

**Figure 4:**
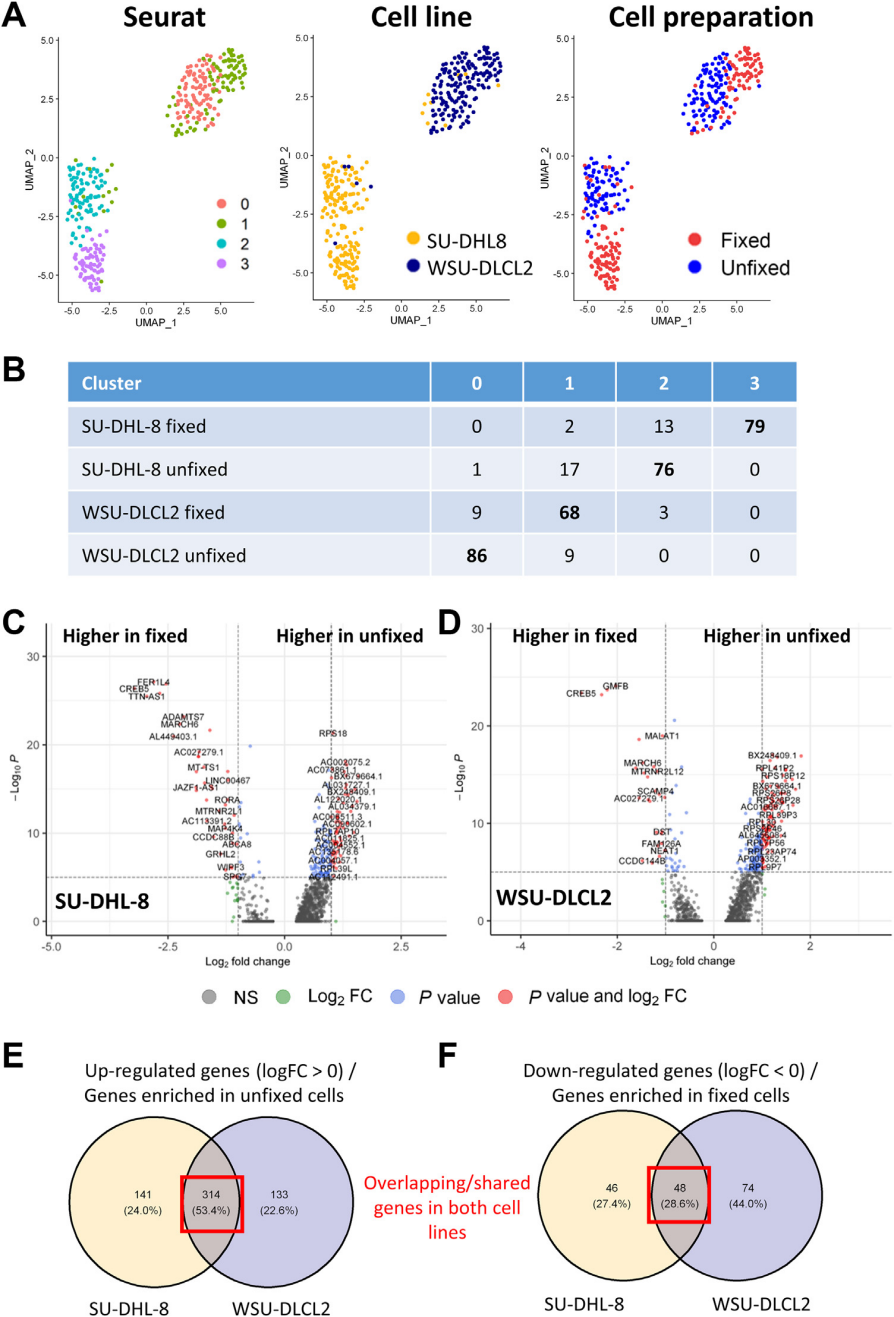
B-cell RNA-sequencing transcriptional profiles segregate based on cell biology and not cell preparation method. **A:** Uniform Manifold Approximation and Projection (UMAP) plots showing cells from two diffuse large B-cell lymphoma cell lines clustering according to Seurat, cell line, and cell preparation method. **B:** Cell numbers associated with clusters outlined in **A**; boldfaced values indicate the cell type most prevalent in that cluster. **C** and **D:** Volcano plots showing differential gene expression between fixed and unfixed cells for SU-DHL-8 (**C**) and WSU-DLCL2 (**D**) cell lines. **E** and **F:** Venn diagrams identifying (up-regulated) genes enriched in unfixed cell lines (**E**) and (down-regulated) genes enriched in fixed cells (**F**). FC, fold change; NS, not significant.

Unsupervised clustering analysis identified four clusters that primarily separated on cell of origin and second, to a lesser extent, on sample preparation method (fixed or unfixed) (Figure 4A). Segregation of clusters 0 and 1 (largely composed of WSU-DLCL2 cells) and clusters 2 and 3 (from SU-DHL-8 cells) (Figure 4B) was fundamentally associated with highly expressed genes discriminating the biology of these two cells lines (Supplemental Figure S4A). For example, WSU-DLCL2 cells displayed enrichment for genes associated with the cluster marker CD20 (*MS4A1*) and germinal center formation, whereas SU-DHL-8 cells expressed enhanced levels of genes associated with proliferation and survival (Supplemental Figure S4A and Supplemental Table S4). Elevated BCL2 expression was also detected in the t(14;18)+ WSU-DLCL2 cell line (Supplemental Figure S4B).

One important observation was that the expression levels of genes, across the clusters discriminating B-cell biology, appear unaffected by fixation (Supplemental Figure S4A). This was also apparent for the most abundantly expressed genes, expressed in both cell lines (Supplemental Figure S4C). To confirm this, the transcriptional phenotypes associated with cell preparation were characterized. The scRNA-seq data series was analyzed to identify notably enriched pathways that could discriminate between live and fixed cells, and potentially generate a fixation signature. In total, 549 and 569 differentially expressed genes were identified between unfixed and glyoxal-fixed (G-E) cells for SU-DHL-8 and WSU-DLCL2 cell lines, respectively (adjusted *P* < 0.05) (Figure 4, C and D, and Supplemental Table S5). Genes up-regulated (or enriched) in unfixed cells were found to be associated with ribosomal protein pseudogenes (SU-DHL-8: 455 genes; and WSU-DLCL2: 447 genes) (Supplemental Table S5), whereas down-regulated genes, or genes enriched in fixed cells, were primarily linked to a small number of genes involved in transcriptional regulation (SU-DHL-8: 94 genes; and WSU-DLCL2: 122 genes) (Supplemental Table S5). Of these differentially expressed genes, almost half (362 genes, 47.9%) were shared between the two cells lines (314 enriched in unfixed cells and 48 enriched in fixed cells) (Figure 4, E and F); however; no significant pathway enrichment was detected in fixed cells (data not shown).

Overall, the clustering patterns outlined in Figure 4A, and subsequent differential gene expression profiles, suggest that fixed and unfixed cells have more overlapping or common transcriptional characteristics than those shared between the two cell lines.

## Discussion

Characterization of residual disease within a heterogeneous cell population or tissue remains a challenge, particularly as current methods focusing on droplet-based technologies fail to capture unique or rare cell subtypes, when sample yield is limited. Enrichment for such populations, predominantly when the biomarker of interest is localized intracellularly, provides an additional layer of complexity for downstream sequencing applications. Within this study, successful enrichment for malignant cells, through intracellular fluorescent antibody detection, was demonstrated using B-cell lymphoma as a paradigm to test this technique. The presence of t(14;18) chromosomal translocation, a key genetic event in several B-cell lymphomas, suggests that the resulting overexpression of BCL2 can be potentially used as a unique biomarker to distinguish and enrich malignant cells. To test this hypothesis, different flow-based methods, all requiring prior fix and permeabilization of cells, were compared to detect intracellular BCL2 expression, while maximizing RNA integrity for use in downstream single-cell sequencing applications.

Initial fixing steps, particularly when using reagents like paraformaldehyde, can induce stress responses that affect the transcriptome, causing partial degradation. In recent years, significant improvements to fixing protocols have not only led to enhanced sample preservation, but in terms of biology, advances in the characterization of cellular subtypes and identifying molecular signatures of tumor cells (particularly when substituting for methanol-based cell fixation procedures).^18^^,^^26^, ^27^, ^28^, ^29^, ^30^ One main advantage of alcohol-based fixatives is the simultaneous permeabilization of cell membranes, enabling intracellular staining; conversely, a major limitation of this step is that it allows extracellular and intra-organellar ribonucleases to enter the cytoplasm and degrade RNA. From the data presented within, the extent of degradation varied between experimental conditions (ie, fixatives used) and between cell types and tissue samples. Overall, glyoxal fixation demonstrated superior results in terms of the minimal effects on surface and intracellular expression, and RNA quality, compared with the alternative fixatives evaluated. Furthermore, G-E profiles were comparable with live-sorted cells in both bulk mRNA-seq and scRNA-seq analyses, demonstrating the potential of this workflow for characterizing malignant cells, following fixation.

Within the present study, malignant cells were successfully enriched using a fluorescent-conjugated antibody to detect an intracellular target. Furthermore, overexpression of BCL2, resulting from the t(14;18) chromosomal translocation in lymphomas, such as follicular lymphoma, can be used as a surrogate marker to distinguish, and enrich for, tumor cells. Equally, in Burkitt lymphoma, mantle cell lymphoma, and diffuse large B-cell lymphoma, where the mechanisms of chromosomal translocation are similar [ie, resulting from errors in V(D)J recombination],^31^ overexpression of tumor-specific oncogenes MYC (MYC proto-oncogene), CCND1 (cyclin D1), and BCL6 (BCL6 transcription repressor) could also be targeted using this enrichment strategy. Furthermore, the scope for such a workflow is not limited to hematological malignancies. The cellenONE F1.4 can facilitate isolation of different cell sizes and morphologies, and when combined with single-cell transcriptomic analysis, has potential to study residual disease in a pan-cancer setting.^32^

Finally, our workflow offers an attractive, and cost-effective, alternative to high-throughput, droplet-based methods, when cellular input is low or requires prior enrichment to detect rare populations, such as MRD. MRD is likely heterogeneous, containing a mix of mature and precursor populations that serve as a substrate for tumor relapse. In-depth characterization of MRD and precursor populations in FL^16^^,^^17^^,^^33^, ^34^, ^35^, ^36^ has not been possible to date. This is mainly due to difficulties in detecting and isolating low-level residual cells, especially in the peripheral blood following anti-CD20 targeted therapy (such as rituximab and obinutuzumab), where standard laboratory or radiological tests struggle to detect subclinical involvement. However, overexpression of BCL2, combined with the cellenONE F1.4 fluorescence imaging application, could serve to identify tumor cells in cases of MRD for further downstream analyses.

In summary, we have successfully developed a workflow combining fixation and permeabilization, and intracellular staining protocols for detection and isolation of malignant cells, using the cellenONE F1.4 platform. We propose that this application can be widely used to identify biomarkers requiring intracellular detection, to enrich for rare populations. Furthermore, the study demonstrates the effectiveness of this application, particularly in the context of MRD. This method represents an advancement, as it not only detects the presence (like real-time quantitative PCR methods), but also allows for phenotypic (eg, transcriptome) characterization, of residual tumor cells. Through characterization of MRD, a better understanding of the mechanisms driving chemoresistance and relapse will enable effective strategies for early detection, intervention, and eradication of residual clones with relapse potential.

## Supplemental Data

Supplemental Figure S1 Optimizing cell fixing and permeabilization conditions for flow cytometry and RNA-sequencing experiments. **A:** Flow cytometry profiles of B-cell lymphoma 2 [BCL2; phycoerythrin (PE)] intensities for SU-DHL-8 (orange) and WSU-DLCL2 (blue) cell lines, and CD19^+^CD20^+^ follicular lymphoma (FL) patient B cells (red; Patient A). Cells were fixed and permeabilized according to the protocols for the 10x Chromium platform and PrimeFlow assay, as well as from the split pool ligation-based transcriptome sequencing (SPLiT-seq) and previous publications.^18^, ^19^, ^20^**B:** Flow cytometry contour plots for FL patient peripheral blood mononuclear cells (PBMCs) labeled with anti-CD19 (AF-647), CD20 (AF-647), and CD3 (AF-488), fixed/permeabilized according to the conditions outlined above, and analyzed using a BD Fortessa instrument. **C:** RNA integrity was assessed and scored [RNA integrity number (RIN)] in FL unsorted PBMCs and sorted B cells [by fluorescence-activated cell sorting (FACS) and negative selection; Patient A], following fixation and permeabilization of the cells. Paraformaldehyde (PFA)/Triton-X or glyoxal [with ethanol (EtOH)]/methanol fixation/permeabilization conditions were compared with unfixed cells, and RIN scores were generated using the Tape Station (Agilent Technologies, Santa Clara, CA). **D:** Repeated experiment of Figure 2A: RNA integrity was assessed and scored (RIN) in two B-cell lymphoma cell lines (WSU-DLCL2 and SU-DHL-8), and FL B cells (isolated from PBMCs from Patient A, following negative magnetic sorting), and compared across the different fixation and permeabilization conditions. **E:** The panel of fixatives was further tested on a second FL PBMC sample (Patient B) (Supplemental Table S1), in addition to two non–B-cell lymphoma cell lines, an adherent breast adenocarcinoma cell line (MCF-7, previously assessed by Channathodiyil and Houseley^20^), and an acute myeloid leukemia cell line (OCI-AML-3). Consistent with the results in **D**, RIN values were superior with glyoxal (without ethanol) across the samples, when compared with other fixatives.

Supplemental Figure S2 B-cell lymphoma 2 (BCL2) overexpression can be used as a biomarker to distinguish t(14;18)+ from t(14;18)– B cells. **A:** DNA was extracted from fluorescence-activated cell sorted (FACS) BCL2 high-expressing cells (following the glyoxal without ethanol fixed cell preparation method), originating from serial dilutions containing ≤1% WSU-DLCL2 t(14;18)+ cell line in a t(14;18)– SU-DHL-8 background. PCR analysis detected BCL2 [major breakpoint region (MBR)]/J(H) rearrangement only in the BCL2 high-expressing cell fraction. **B:** Sorting protocol according to BCL2 expression: primary follicular lymphoma (FL) peripheral blood mononuclear cells (PBMCs; from Patient A) (Supplemental Table S1) were diluted in a healthy PBMC sample at 1% and 0.1% of the PBMC total. A minimum of 10,000 CD19^+^CD20^+^ B cells were then FACS into 1.5-mL tubes, based on high, intermediate (medium), and low BCL2 expression; gates were set to include 10% of events within each subpopulation. DNA extracted for MBR detection by digital-droplet PCR (ddPCR).^21^ Samples were analyzed together with the human B-cell lymphoma cell line DoHH2 as positive control and the reaction mixture with no DNA as a negative control. FSC, forward scatter.

Supplemental Figure S3 Comparison of single-cell (pseudo bulk) and bulk mRNA-sequencing (mRNA-seq) analysis. **A:** Correlation plots showing a positive association between the genes expressed in cell lines from single-cell and bulk RNA-seq sets. **B** and **C:** Heat maps generated from the top 100 differentially expressed genes (Supplemental Table S3) show strong correlation between bulk mRNA samples and single-cell pseudo bulk samples [fixed (**B**) and unfixed (**C**) cells]. scRNA-seq, single-cell RNA sequencing.

Supplemental Figure S4 Uniform Manifold Approximation and Projection (UMAP) plots of co-expressed and differentially expressed genes between cell lines. **A:** Expression of genes defining the cell line biology [WSU-DLCL2 (**top panels**) and SU-DHL8 (**bottom panels**)]; cell positions are from the UMAP plots in Figure 2A. **B:** B-cell lymphoma 2 (BCL2) expression was highest in t(14;18)+ WSU-DLCL2 cells, across both fixed and unfixed cells, compared with in t(14;18)– SU-DHL-8 cells. **C:** UMAP plots demonstrating the highest-expressing genes in both cell lines are unaffected by cell preparation method.

## References

[1] Luskin M.R., Murakami M.A., Manalis S.R., Weinstock D.M. (2018). Targeting minimal residual disease: a path to cure?. Nat Rev Cancer.

[2] Ben Lassoued A., Nivaggioni V., Gabert J. (2014). Minimal residual disease testing in hematologic malignancies and solid cancer. Expert Rev Mol Diagn.

[3] Scherer F., Kurtz D.M., Diehn M., Alizadeh A.A. (2017). High-throughput sequencing for noninvasive disease detection in hematologic malignancies. Blood.

[4] Peng Y., Mei W., Ma K., Zeng C. (2021). Circulating tumor DNA and minimal residual disease (MRD) in solid tumors: current horizons and future perspectives. Front Oncol.

[5] Tivey A., Church M., Rothwell D., Dive C., Cook N. (2022). Circulating tumour DNA - looking beyond the blood. Nat Rev Clin Oncol.

[6] Wang X., He Y., Zhang Q., Ren X., Zhang Z. (2021). Direct comparative analyses of 10X genomics chromium and smart-seq2. Genomics Proteomics Bioinformatics.

[7] Andor N., Simonds E.F., Czerwinski D.K., Chen J., Grimes S.M., Wood-Bouwens C., Zheng G.X.Y., Kubit M.A., Greer S., Weiss W.A., Levy R., Ji H.P. (2019). Single-cell RNA-Seq of follicular lymphoma reveals malignant B-cell types and coexpression of T-cell immune checkpoints. Blood.

[8] Gao C., Zhang M., Chen L. (2020). The comparison of two single-cell sequencing platforms: BD rhapsody and 10x genomics chromium. Curr Genomics.

[9] Roider T., Seufert J., Uvarovskii A., Frauhammer F., Bordas M., Abedpour N., Stolarczyk M., Mallm J.P., Herbst S.A., Bruch P.M., Balke-Want H., Hundemer M., Rippe K., Goeppert B., Seiffert M., Brors B., Mechtersheimer G., Zenz T., Peifer M., Chapuy B., Schlesner M., Müller-Tidow C., Fröhling S., Huber W., Anders S., Dietrich S. (2020). Dissecting intratumour heterogeneity of nodal B-cell lymphomas at the transcriptional, genetic and drug-response levels. Nat Cell Biol.

[10] Han G., Den Q., Marques-Piubelli M.L., Dai E., Dang M., Ma M.C.J., Li X., Yang H., Henderson J., Kudryashova O., Meerson M., Isaev S., Kotlov N., Nomie K.J., Bagaev A., Parra E.R., Solis Soto L.M., Parmar S., Hagemeister F.B., Ahmed S., Iyer S.P., Samaniego F., Steiner R., Fayad L., Lee H., Fowler N.H., Flowers C.R., Strati P., Westin J.R., Neelapu S.S., Nastoupil L.J., Vega F., Wang L., Green M.R. (2022). Follicular lymphoma microenvironment characteristics associated with tumor cell mutations and MHC class II expression. Blood Cancer Discov.

[11] Ziegenhain C., Vieth B., Partekh S., Reinius B., Guillaumet-Adkins A., Smets M., Leonhardt H., Heyn H., Hellmann I., Enard W. (2017). Comparative analysis of single-cell RNA sequencing methods. Mol Cell.

[12] Bagnoli J.W., Ziegenhain C., Janjic A., Wange L.E., Vieth B., Parekh S., Geuder J., Hellmann I., Enard W. (2018). Sensitive and powerful single-cell RNA sequencing using mcSCRB-seq. Nat Commun.

[13] Ladetto M., Tavarozzi R., Pott C. (2020). Minimal residual disease in mantle cell lymphoma: methods and clinical significance. Hematol Oncol Clin North Am.

[14] Galimberti S., Genuardi E., Mazziotta F., Iovino L., Morabito F., Grassi S., Ciabatti E., Guerrini F., Petrini M. (2019). The minimal residual disease in non-hodgkin's lymphomas: from the laboratory to the clinical practice. Front Oncol.

[15] Gritti G., Pavoni C., Rambaldi A. (2017). There a role for minimal residual disease monitoring in follicular lymphoma in the chemo-immunotherapy era?. Mediterr J Hematol Infect Dis.

[16] Ladetto M., Lobetti-Bodoni C., Mantoan B., Ceccarelli M., Boccomini C., Genuardi E., Chiappella A., Baldini L., Rossi G., Pulsoni A., Di Raimondo F., Rigacci L., Pinto A., Galimberti S., Bari A., Rota-Scalabrini D., Ferrari A., Zaja F., Gallamini A., Specchia G., Musto P., Rossi F.G., Gamba E., Evangelista A., Vitolo U., Fondazione Italiana Linfomi (2013). Persistence of minimal residual disease in bone marrow predicts outcome in follicular lymphomas treated with a rituximab-intensive program. Blood.

[17] Pott C., Wellnitz D., Ladetto M. (2021). Minimal residual disease in follicular lymphoma. Ann Lymphoma.

[18] Chen J., Cheung F., Shi R., Zhou H., Lu W., CHI Consortium (2018). PBMC fixation and processing for chromium single-cell RNA sequencing. J Transl Med.

[19] Rosenberg A.B., Roco C.M., Muscat R.A., Kuchin A., Sample P., Yao Z., Graybuck L.T., Peeler D.J., Mukherjee S., Chen W., Pun S.H., Sellers D.L., Tasic B., Seelig G. (2018). Single-cell profiling of the developing mouse brain and spinal cord with split-pool barcoding. Science.

[20] Channathodiyil P., Houseley J. (2021). Glyoxal fixation facilitates transcriptome analysis after antigen staining and cell sorting by flow cytometry. PLoS One.

[21] Drandi D., Kubiczkova-Besse L., Ferrero S., Dani N., Passera R., Mantoan B., Gambella M., Monitillo L., Saraci E., Ghione P., Genuardi E., Barbero D., Omedè P., Barberio D., Hajek R., Vitolo U., Palumbo A., Cortelazzo S., Boccadoro M., Inghirami G., Ladetto M. (2015). Minimal residual disease detection by droplet digital PCR in multiple myeloma, mantle cell lymphoma, and follicular lymphoma: a comparison with real-time PCR. J Mol Diagn.

[22] Kim D., Paggi J.M., Park C., Bennet C., Salzberg S.L. (2019). Graph-based genome alignment and genotyping with HISAT2 and HISAT-genotype. Nat Biotechnol.

[23] Anders S., Pyl P.T., Huber W. (2015). HTSeq--a Python framework to work with high-throughput sequencing data. Bioinformatics.

[24] Parekh S., Ziegenhaim C., Vieth B., Enard W., Hellmann I. (2018). zUMIs - a fast and flexible pipeline to process RNA sequencing data with UMIs. Gigascience.

[25] Choudhary S., Satija R. (2022). Comparison and evaluation of statistical error models for scRNA-seq. Genome Biol.

[26] Wang X., Yu L., Wu A.R. (2021). The effect of methanol fixation on single-cell RNA sequencing data. BMC Genomics.

[27] Thomsen E.R., Mich J.K., Yao Z., Hodge R.D., Doyle A.M., Jang S., Shehata S.I., Nelson A.M., Shapovalova N.V., Levi B.P., Ramanathan S. (2016). Fixed single-cell transcriptomic characterization of human radial glial diversity. Nat Methods.

[28] Katzenelenbogen Y., Sheban F., Yalin A., Yofe I., Svetlichnyy D., Jaitin D.A., Bornstein C., Moshe A., Keren-Shaul H., Cohen M., Wang S.Y., Li B., David E., Salame T.M., Weiner A., Amit I. (2020). Coupled scRNA-seq and intracellular protein activity reveal an immunosuppressive role of TREM2 in cancer. Cell.

[29] Phan H.V., van Gent M., Drayman N., Basu A., Gack M.U., Tay S. (2021). High-throughput RNA sequencing of paraformaldehyde-fixed single cells. Nat Commun.

[30] García-Castro H., Kenny N.J., Iglesias M., Álvarez-Campos P., Mason V., Elek A., Schönauer A., Sleight V.A., Neiro J., Aboobaker A., Permanyer J., Irimia M., Sebé-Pedrós A., Solana J. (2021). ACME dissociation: a versatile cell fixation-dissociation method for single-cell transcriptomics. Genome Biol.

[31] Zheng J. (2013). Oncogenic chromosomal translocations and human cancer. Oncol Rep.

[32] Shomroni O., Sitte M., Schmidt J., Parbin S., Ludewig F., Yigit G., Zelarayan L.C., Streckfuss-Bömeke K., Wollnik B., Salinas G. (2022). A novel single-cell RNA-sequencing approach and its applicability connecting genotype to phenotype in ageing disease. Sci Rep.

[33] Okosun J., Bödör C., Wang J., Araf S., Yang C.Y., Pan C., Boller S., Cittaro D., Bozek M., Iqbal S., Matthews J., Wrench D., Marzec J., Tawana K., Popov N., O'Riain C., O'Shea D., Carlotti E., Davies A., Lawrie C.H., Matolcsy A., Calaminici M., Norton A., Byers R.J., Mein C., Stupka E., Lister T.A., Lenz G., Montoto S., Gribben J.G., Fan Y., Grosschedl R., Chelala C., Fitzgibbon J. (2014). Integrated genomic analysis identifies recurrent mutations and evolution patterns driving the initiation and progression of follicular lymphoma. Nat Genet.

[34] Carbone A., Roulland S., Gloghini A., Younes A., von Keudell G., López-Guillermo A., Fitzgibbon J. (2019). Follicular lymphoma. Nat Rev Dis Primers.

[35] Weigert O., Kopp N., Lane A.A., Yoda A., Darhlberg S.E., Neuberg D., Bahar A.Y., Chapuy B., Kutok J.L., Longtine J.A., Kuo F.C., Haley T., Salois M., Sullivan T.J., Fisher D.C., Fox E.A., Rodig S.J., Antin J.H., Weinstock D.M. (2012). Molecular ontogeny of donor-derived follicular lymphomas occurring after hematopoietic cell transplantation. Cancer Discov.

[36] Summers K.E., Goff L.K., Wilson A.G., Gupta R.K., Lister T.A., Fitzgibbon J. (2001). Frequency of the Bcl-2/IgH rearrangement in normal individuals: implications for the monitoring of disease in patients with follicular lymphoma. J Clin Oncol.

