# Supplementary figures and images for: Defining an Optimized Workflow for Enriching and Analyzing Residual Tumor Populations Using Intracellular Markers
Source: J Mol Diagn. 2024 Jan 26;26(4):245–56. doi: 10.1016/j.jmoldx.2024.01.003 (PMC12178326; doi:10.1016/j.jmoldx.2024.01.003)

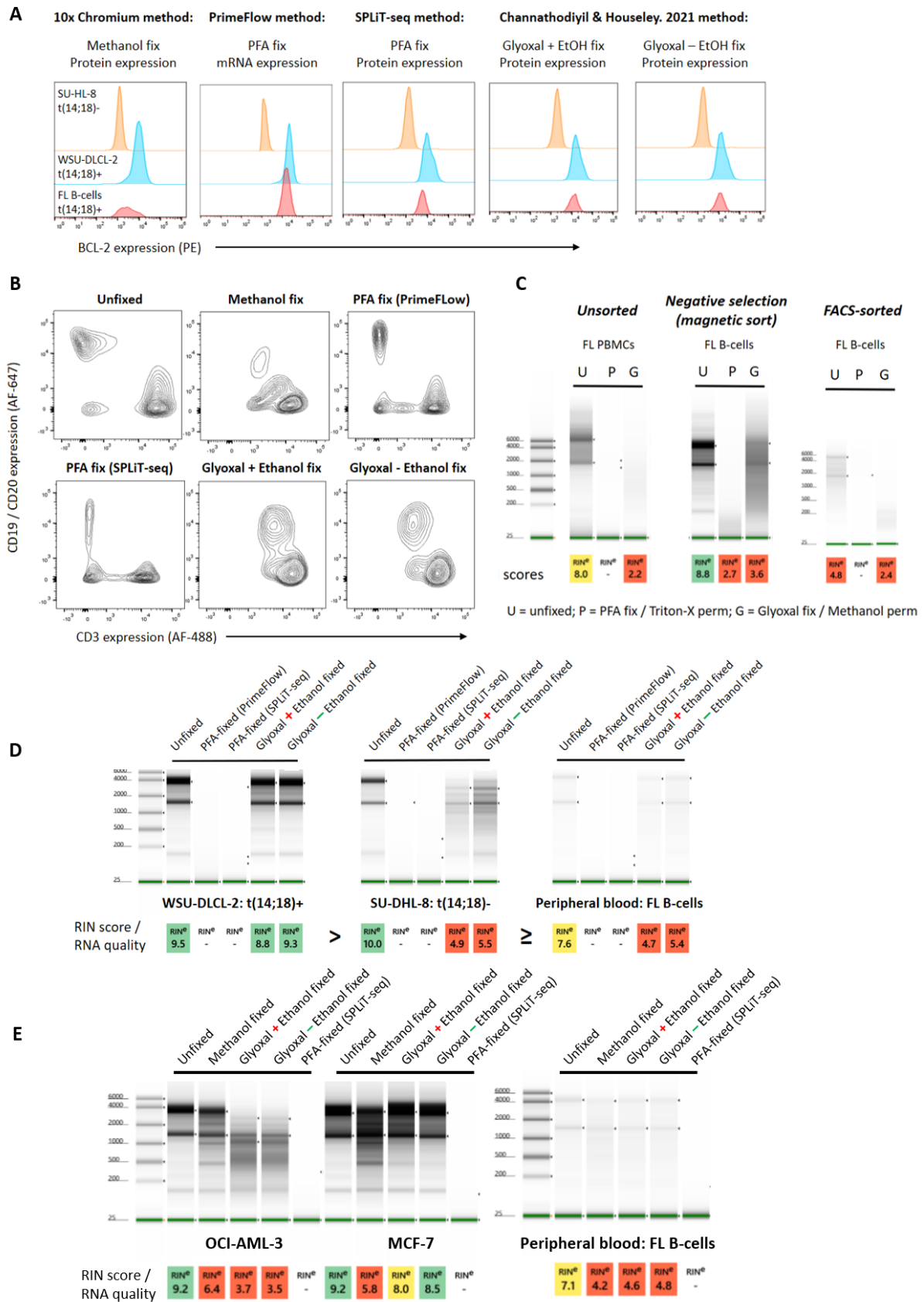

Supplement: Supplemental Figure S1 — Optimizing cell fixing and permeabilization conditions for flow cytometry and RNA-sequencing experiments. A: Flow cytometry profiles of B-cell lymphoma 2 [BCL2; phycoerythrin (PE)] intensities for SU-DHL-8 (orange) and WSU-DLCL2 (blue) cell lines, and CD19+CD20+ follicular lymphoma (FL) patient B cells (red; Patient A). Cells were fixed and permeabilized according to the protocols for the 10x Chromium platform and PrimeFlow assay, as well as from the split pool ligation-based transcriptome sequencing (SPLiT-seq) and previous publications.18, 19, 20B: Flow cytometry contour plots for FL patient peripheral blood mononuclear cells (PBMCs) labeled with anti-CD19 (AF-647), CD20 (AF-647), and CD3 (AF-488), fixed/permeabilized according to the conditions outlined above, and analyzed using a BD Fortessa instrument. C: RNA integrity was assessed and scored [RNA integrity number (RIN)] in FL unsorted PBMCs and sorted B cells [by fluorescence-activated cell sorting (FACS) and negative selection; Patient A], following fixation and permeabilization of the cells. Paraformaldehyde (PFA)/Triton-X or glyoxal [with ethanol (EtOH)]/methanol fixation/permeabilization conditions were compared with unfixed cells, and RIN scores were generated using the Tape Station (Agilent Technologies, Santa Clara, CA). D: Repeated experiment of Figure 2A: RNA integrity was assessed and scored (RIN) in two B-cell lymphoma cell lines (WSU-DLCL2 and SU-DHL-8), and FL B cells (isolated from PBMCs from Patient A, following negative magnetic sorting), and compared across the different fixation and permeabilization conditions. E: The panel of fixatives was further tested on a second FL PBMC sample (Patient B) (Supplemental Table S1), in addition to two non–B-cell lymphoma cell lines, an adherent breast adenocarcinoma cell line (MCF-7, previously assessed by Channathodiyil and Houseley20), and an acute myeloid leukemia cell line (OCI-AML-3). Consistent with the results in D, RIN values were superior with glyo [file mmc1.pdf]

**A** WSU-DLCL-2 spike-in to SU-DHL-8 background

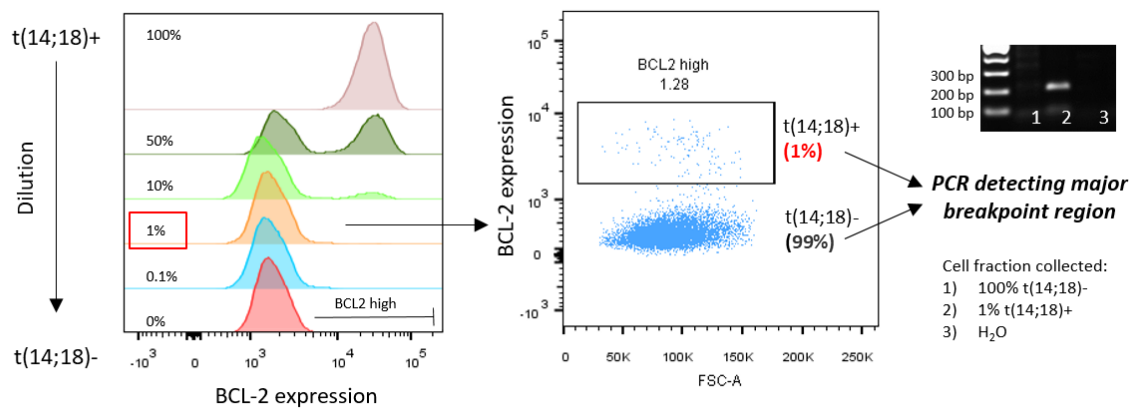

**B** FL PBMC spike-in to healthy PBMC background

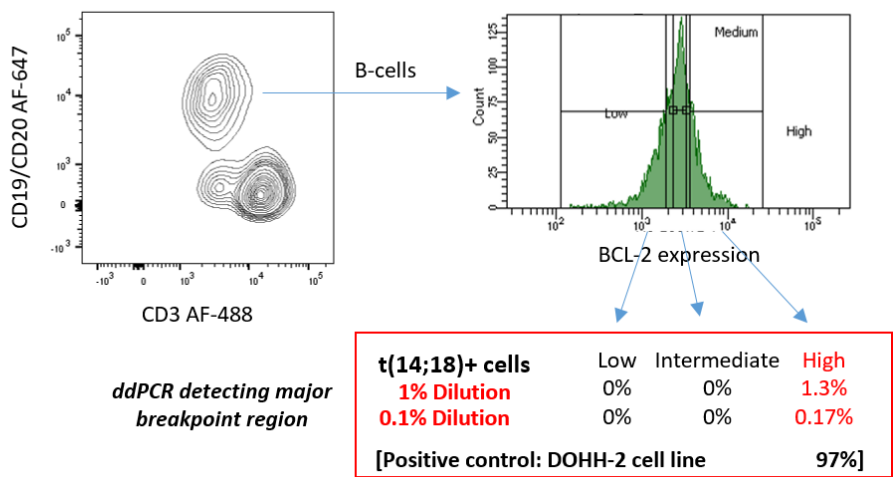

Supplement: Supplemental Figure S2 — B-cell lymphoma 2 (BCL2) overexpression can be used as a biomarker to distinguish t(14;18)+ from t(14;18)– B cells. A: DNA was extracted from fluorescence-activated cell sorted (FACS) BCL2 high-expressing cells (following the glyoxal without ethanol fixed cell preparation method), originating from serial dilutions containing ≤1% WSU-DLCL2 t(14;18)+ cell line in a t(14;18)– SU-DHL-8 background. PCR analysis detected BCL2 [major breakpoint region (MBR)]/J(H) rearrangement only in the BCL2 high-expressing cell fraction. B: Sorting protocol according to BCL2 expression: primary follicular lymphoma (FL) peripheral blood mononuclear cells (PBMCs; from Patient A) (Supplemental Table S1) were diluted in a healthy PBMC sample at 1% and 0.1% of the PBMC total. A minimum of 10,000 CD19+CD20+ B cells were then FACS into 1.5-mL tubes, based on high, intermediate (medium), and low BCL2 expression; gates were set to include 10% of events within each subpopulation. DNA extracted for MBR detection by digital-droplet PCR (ddPCR).21 Samples were analyzed together with the human B-cell lymphoma cell line DoHH2 as positive control and the reaction mixture with no DNA as a negative control. FSC, forward scatter. [file mmc2.pdf]

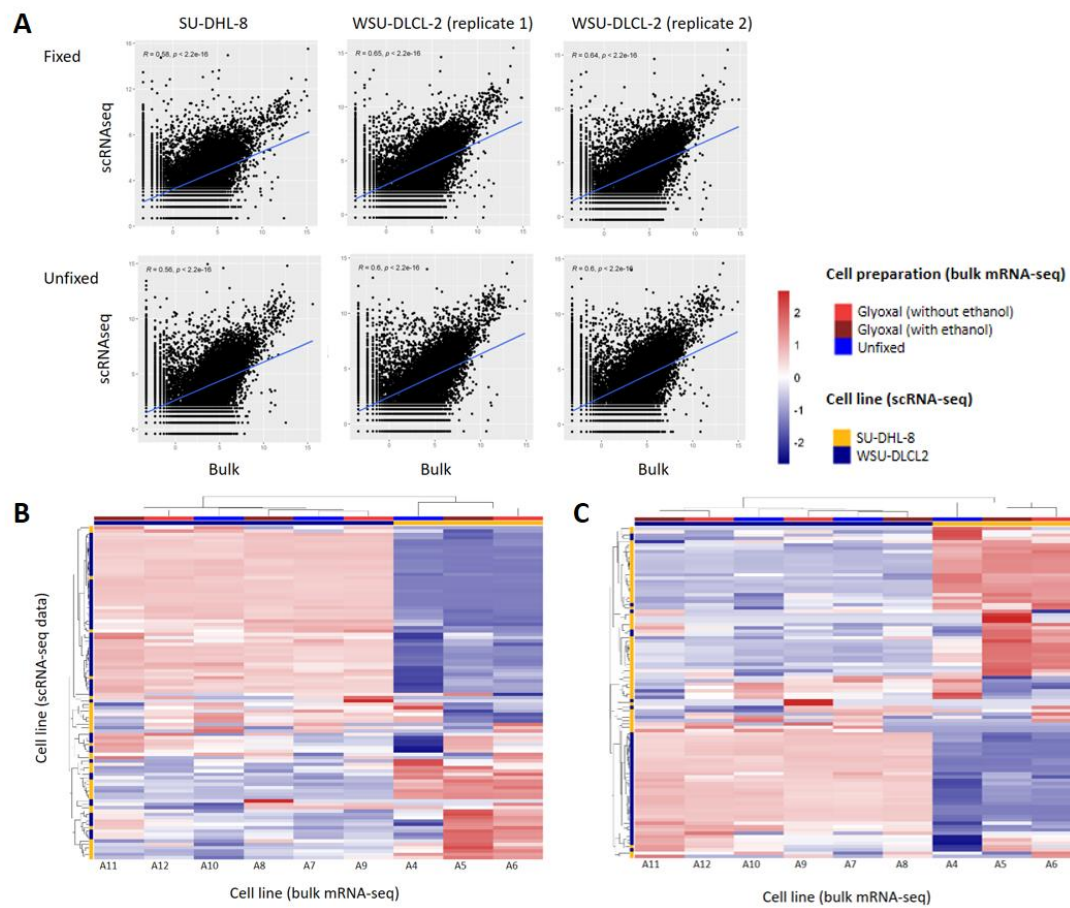

Supplement: Supplemental Figure S3 — Comparison of single-cell (pseudo bulk) and bulk mRNA-sequencing (mRNA-seq) analysis. A: Correlation plots showing a positive association between the genes expressed in cell lines from single-cell and bulk RNA-seq sets. B and C: Heat maps generated from the top 100 differentially expressed genes (Supplemental Table S3) show strong correlation between bulk mRNA samples and single-cell pseudo bulk samples [fixed (B) and unfixed (C) cells]. scRNA-seq, single-cell RNA sequencing. [file mmc3.pdf]

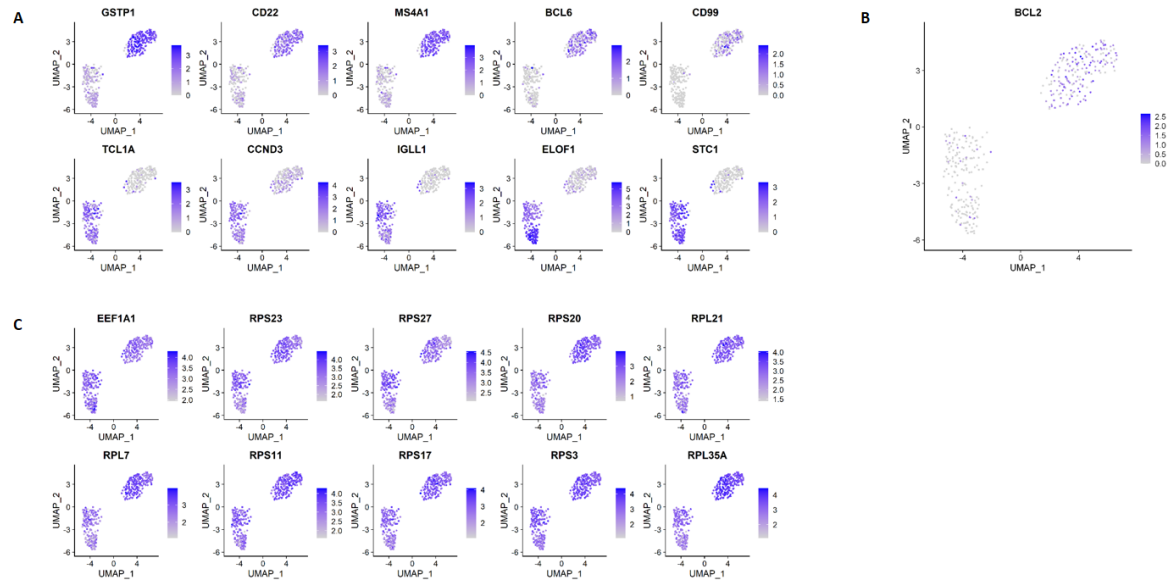

Supplement: Supplemental Figure S4 — Uniform Manifold Approximation and Projection (UMAP) plots of co-expressed and differentially expressed genes between cell lines. A: Expression of genes defining the cell line biology [WSU-DLCL2 (top panels) and SU-DHL8 (bottom panels)]; cell positions are from the UMAP plots in Figure 2A. B: B-cell lymphoma 2 (BCL2) expression was highest in t(14;18)+ WSU-DLCL2 cells, across both fixed and unfixed cells, compared with in t(14;18)– SU-DHL-8 cells. C: UMAP plots demonstrating the highest-expressing genes in both cell lines are unaffected by cell preparation method. [file mmc4.pdf]
